# Impact of positive lymph nodes and RAI therapy on survival in N1b papillary thyroid carcinoma

**DOI:** 10.3389/fendo.2025.1551075

**Published:** 2025-05-22

**Authors:** Jie Jian, Meng Wei, Xumei Li, Qian Xiong, Jiangming Xiang, Shengping Zhao, Yuxi Peng, Jingjing Huang

**Affiliations:** ^1^ Department of Pathology, Chongqing Changshou District Maternal and Child Health Hospital, Chongqing, China; ^2^ Department of Surgery, Chongqing Changshou District Maternal and Child Health Hospital, Chongqing, China; ^3^ Department of Pathology, Huai’an Maternal and Child Health Care Center of Jiangsu Province Affiliated Hospital of Yangzhou University, Huai'an, China; ^4^ Department of Pathology, Bo’ai Hospital of Zhongshan, Zhongshan, China

**Keywords:** papillary thyroid carcinoma, positive lymph node, radioactive iodine, survival, SEER

## Abstract

**Background:**

Patients with N1b papillary thyroid carcinoma (PTC) was associated with a worse prognosis. The prognostic role of positive lymph nodes (PLN) and whether postoperative radioactive iodine (RAI) therapy conferred a survival benefit were debatable issues in these patients.

**Methods:**

Data were drawn from the SEER database for PTC patients with clinical N1b disease diagnosed between 2004-2015. All patient underwent total thyroidectomy with or without RAI. Patients were categorized by age (≥55 years and <55 years) and analyzed based on PLN. Propensity score matching (PSM) were used to balance characteristics between patients who did and did not receive RAI therapy. Overall survival (OS) was the primary outcome. Kaplan-Meier survival analysis and Cox analysis were performed.

**Results:**

A total of 4343 N1b PTC patients were included, with 884 patients aged ≥55 years and 3459 patients aged <55 years. In patients aged ≥55 years, the optimal PLN cutoff for risk stratification was 8. Those with PLN ≥9 had significantly lower 5-year (83.7% vs. 90.1%), 10-year (67.4% vs. 78.8%) and 15-year (50.3% vs. 59.5%) OS rates. After adjusting, the hazard ratio for death in the PLN ≥9 group increased by 30%. After PSM, in subgroup of aged ≥55 years and PLN ≥9, the survival benefit was notable in those received RAI therapy. In contrast, for patients aged ≥55 years and PLN ≤8 or aged <55 years, no survival difference was found between those received RAI and those not.

**Conclusions:**

In N1b PTC patients aged ≥55 years, PLN ≥9 predicted a poorer survival. Postoperative RAI therapy offered survival benefits for this subgroup.

## Introduction

Papillary thyroid carcinoma (PTC) was the most common subtype of thyroid cancer, accounting for approximately 80–85% of all thyroid cancer cases. Its prognosis was generally favorable due to its slow-growing nature and the high rate of curability with appropriate treatment (1). However, a subset of PTC patients presented with cervical lymph node metastases (LNM), which complicated prognosis and treatment strategies. Specifically, patients with clinical N1b disease, characterized by lateral neck LNM, often experienced a more aggressive disease course (2). Epidemiological data highlighted that around 15–30% of PTC patients presented with N1b disease, and these patients tended to have poorer survival outcomes compared to those with N0 or N1a disease, contributing to a substantial subset of patients requiring more aggressive management (3). The 10-year survival rate for patients with N1b disease was significantly lower, approximately 85% for older patients compared to nearly 95% for N0 and N1a cases (4, 5).

There were considerable debates in clinical practice regarding the optimal management strategies of patients with N1b PTC. The American Thyroid Association (ATA) guidelines highlight lymph node involvement as a critical factor in determining the prognosis of these patients (6). Therefore, more aggressive treatment strategies may be required to reduce the risk of recurrence and improve survival outcomes, such as total thyroidectomy followed by radioactive iodine (RAI) therapy. However, clinical studies investigating the role of RAI therapy in N1b patients had yielded conflicting results. Some studies showed that RAI therapy improved survival in N1b patients, particularly in the presence of multiple or extranodal-positive lymph nodes, while the benefit was not universally observed, especially in low-risk or younger patients (4, 7–9). Additionally, age remained an important prognostic factor in thyroid cancer, with younger patients generally exhibiting a more favorable prognosis. According to the ATA guidelines, patients aged 55 years or older were at a higher risk of recurrence and might require more aggressive postoperative treatment (6). Thus, age can influence both prognosis and the decision to proceed with RAI therapy, complicating clinical decision-making in N1b PTC patients.

The number of positive lymph nodes (PLN) was a critical prognostic factor influencing survival outcomes in PTC patients and played a significant role in guiding postoperative treatment decisions. Studies consistently show that a higher number of PLN was correlated with poorer outcomes in PTC patients, reinforcing its importance as a marker for risk stratification (10, 11). However, the precise cutoff for PLN remained a topic of ongoing debate, with limited research specifically addressing N1b patients. Identifying the optimal threshold for PLN and elucidating its role in predicting patient survival outcomes could significantly enhance the clinical management of this high-risk subgroup.

Given these unresolved issues, this study aimed to investigate the prognostic role of PLN in patients with N1b PTC and explored whether postoperative RAI therapy conferred a survival benefit in this specific group. By identifying the optimal cutoff for PLN and evaluating survival outcomes based on RAI treatment, our goal was to provide clinical evidence for precise managing N1b PTC patients.

## Methods

### Patient screening

Data were obtained from the SEER database, specifically the SEER 17 registries (SEER*Stat, version 8.4.3). The inclusion criteria were as follows: (1) primary tumor located in the thyroid, (2) only one primary tumor, (3) histologically confirmed PTC with histology codes 8050/3, 8052/3, 8130/3, 8260/3, 8340–8344/3, 8450/3, and 8452/3 according to the International Classification of Diseases for Oncology, 3rd Edition, (4) diagnosis between 2004 and 2015, (5) clinical N1b (cN1b) disease without distant metastases (M0), and (6) diagnosis age between 15 and 80 years, as older patients were less likely to undergo aggressive treatments due to comorbidities.

The exclusion criteria included: (1) patients diagnosed through autopsy only, (2) patients with missing survival data, (3) those who did not undergo total thyroidectomy, (4) patients with documented data on less than 10 examined lymph nodes (ELNs) and unknown PLN counts, (5) patients who received external beam radiation therapy, and (6) patients who received systemic therapy.

### Study design

According to the 2015 ATA guidelines, age was a critical factor influencing thyroid cancer prognosis. Therefore, patients were stratified into two cohorts: age ≥55 years and age <55 years. Within each cohort, the optimal cutoff for the number of PLN was determined using the "survival" and "survminer" packages in R software. Based on this cutoff, patients were classified into high-risk and low-risk groups to evaluate survival differences.

**Figure 1:**
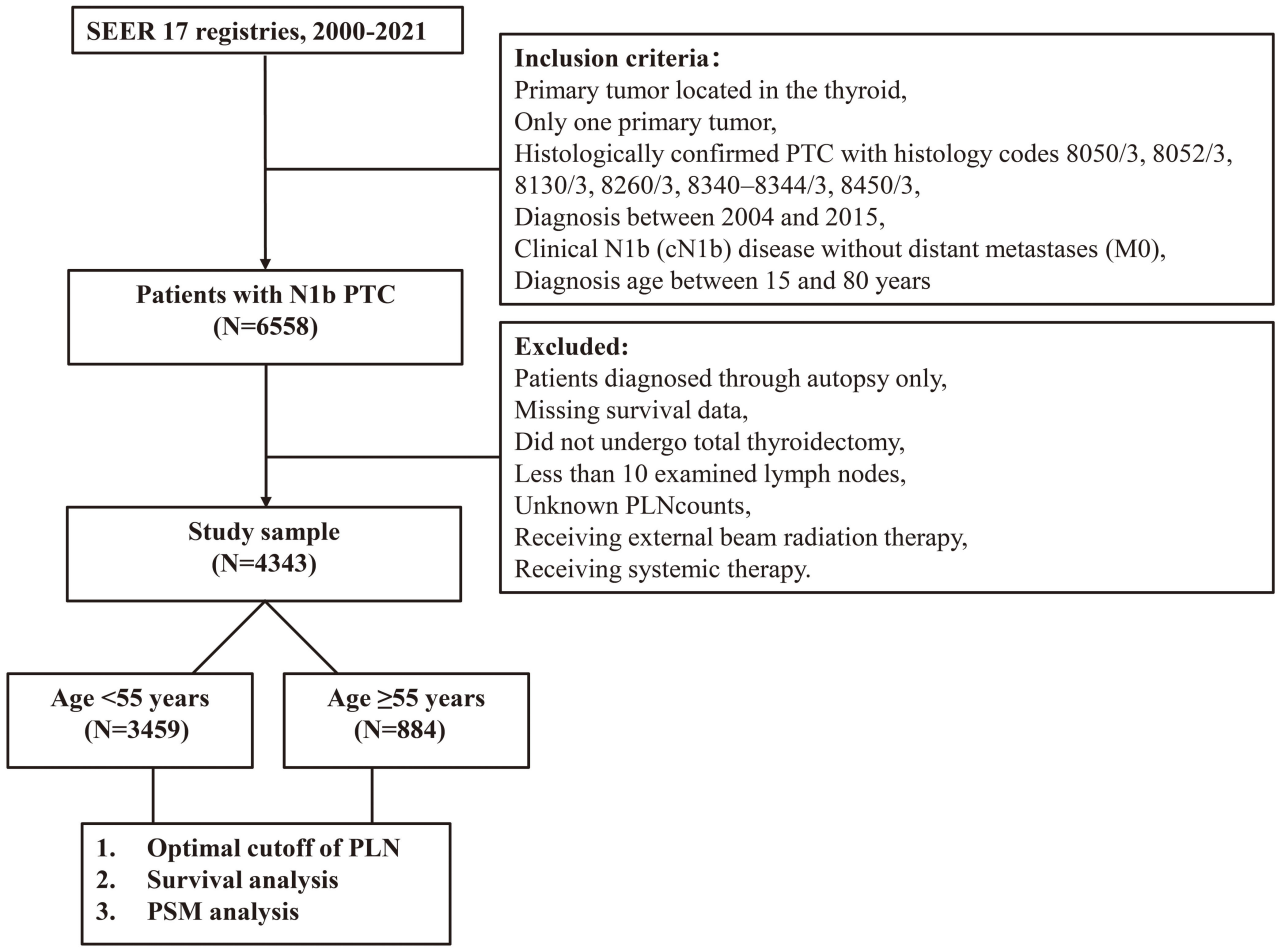
A flowchart to present patients screening and study design.

Propensity score matching (PSM) was subsequently performed to balance baseline characteristics between patients who received postoperative RAI and those who did not. Survival outcomes were then analyzed before and after matching within the high- and low-risk groups. A flowchart of patients screening and study design was presented in **Figure 1**.

### Study outcomes and variables

The primary outcome of this study was overall survival (OS), defined as the time from the date of diagnosis to death from any cause or the last follow-up (December 31, 2021). Key covariates and subgroups included sex (male/female), race (White/Black/Other), tumor size (0–10 mm, 11–20 mm, 21–40 mm, >40 mm, unknown), and RAI therapy status (yes/no).

### Statistical analyses

Clinical characteristics were summarized as counts and percentages, with comparisons performed using Pearson’s Chi-squared test. Survival analyses were conducted using the Kaplan-Meier method and log-rank tests. Five-, 10-, and 15-year survival rates were calculated accordingly. Univariable and multivariable Cox proportional hazard models were used to identify independent risk factors, with hazard ratios (HRs) and 95% confidence intervals (CIs) reported.

PSM was performed to adjust for baseline differences between patients who received RAI therapy and those who did not, using a 1:2 matching ratio.

All statistical analyses were conducted using R software (version 4.4.0; https://www.r-project.org). A two-sided p-value <0.05 was considered statistically significant.

## Results

After screening, a total of 4343 non-metastatic PTC patients with cN1b who underwent total thyroidectomy and had at least 10 ELN were included in this retrospective study. Among these, 884 patients were aged ≥55 years and 3459 were aged <55 years.

### The prognostic role of PLN in patients aged ≥55 years

In patients aged ≥55 years, the optimal cutoff value for PLN to stratify high-risk from low-risk groups was determined to be 8. The clinical characteristics of patients with PLN ≤8 (N =522) and those with PLN ≥9 (N =362) were compared. Patients with PLN ≥9 had a higher proportion of larger tumor sizes. Specifically, 34.8% of patients with PLN ≥9 had tumors in the 21–40 mm range, and 23.8% had tumors >40 mm, compared to 25.9% and 16.3%, respectively, in the PLN ≤8 group (P <0.001). No significant differences in sex, race, or history of RAI therapy were observed (**Table 1**).

**Table 1:** Clinical characteristics of papillary thyroid carcinoma patients aged ≥55 years and comparison stratified by positive lymph nodes.

Variable	All, N =884 (%)	PLN ≤8, N =522 (%)	PLN ≥9, N =362 (%)	P value
Sex				0.179
Male	451 (51.0)	256 (49.0)	195 (53.9)	
Female	433 (49.0)	266 (51.0)	167 (46.1)	
Race				0.230
White	704 (79.6)	424 (81.2)	280 (77.3)	
Black	27 (3.1)	17 (3.3)	10 (2.8)	
Others	153 (17.3)	81 (15.5)	72 (19.9)	
Tumor size, mm				<0.001
0-10	185 (20.9)	143 (27.4)	42 (11.6)	
11-20	238 (26.9)	144 (27.6)	94 (26.0)	
21-40	261 (29.5)	135 (25.9)	126 (34.8)	
>40	171 (19.3)	85 (16.3)	86 (23.8)	
Unknown	29 (3.3)	15 (2.9)	14 (3.9)	
RAI				0.700
No	203 (23.0)	117 (22.4)	86 (23.8)	
Yes	681 (77.0)	405 (77.6)	276 (76.2)	

PLN, positive lymph node; RAI, radioactive iodine.

**Figure 2:**
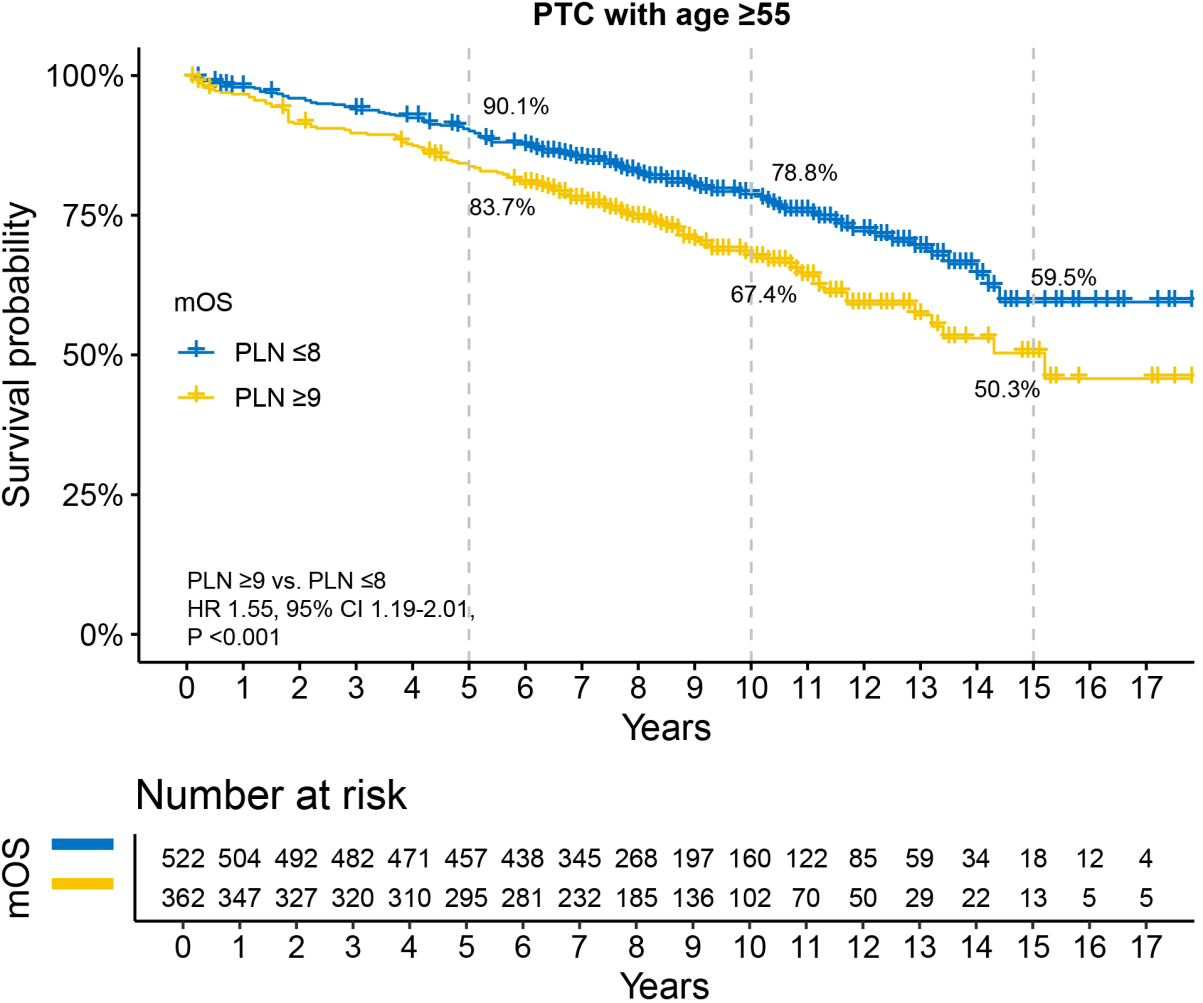
Disparity in survival between PLN ≤8 and PLN ≥9 in N1b PTC patients with age ≥55 years. All patients underwent total thyroidectomy and at least 10 examined lymph nodes. PTC, papillary thyroid carcinoma; PLN, positive lymph node; HR, hazard ratio; CI, confidence interval.

Kaplan-Meier survival analysis revealed that patients with PLN ≤8 had better survival compared to those with PLN ≥9, although the median OS for both groups was not reached (**Figure 2**). The 5-year, 10-year, and 15-year OS rates were 90.1% vs. 83.7%, 78.8% vs. 67.4%, and 59.5% vs. 50.3%, respectively. Univariable and multivariable Cox regression analyses demonstrated that, in addition to PLN, tumor size and RAI therapy were significant predictors of OS (**Table 2**). After adjusting for tumor size and RAI therapy, the HR for death increased by 30% in patients with PLN ≥9 (HR 1.30, 95% CI 1.01–1.70, P =0.049).

**Table 2:** Univariable and multivariable Cox analyses for risk factors associated with overall survival in patients aged ≥55 years.

Variable	Univariable		Multivariable	
	HR (95% CI)	P value	HR (95% CI)	p value
Sex				
Male	Reference	–		
Female	0.81 (0.62-1.05)	0.112		
Race				
White	Reference	–		
Black	1.04 (0.46-2.34)	0.931		
Others	1.14 (0.82-1.59)	0.437		
Tumor size, mm				
0-10	Reference	–	Reference	–
11-20	1.64 (1.02-2.64)	0.041	1.66 (1.03-2.69)	0.038
21-40	1.95 (1.24-3.07)	0.004	1.93 (1.21-3.07)	0.006
>40	3.80 (2.42-5.95)	<0.001	3.79 (2.39-6.02)	<0.001
Unknown	4.51 (2.39-8.53)	<0.001	4.26 (2.24-8.10)	<0.001
RAI				
No	Reference	–	Reference	–
Yes	0.73 (0.55-0.98)	0.037	0.65 (0.48-0.87)	0.004
PLN				
≤8	Reference	–	Reference	–
≥9	1.55 (1.19-2.01)	0.001	1.30 (1.01-1.70)	0.049

HR, hazard ratio; CI, confidence interval; RAI, radioactive iodine; PLN, positive lymph node.

### Survival benefit of RAI in the subgroup of PLN ≥9

**Figure 3:**
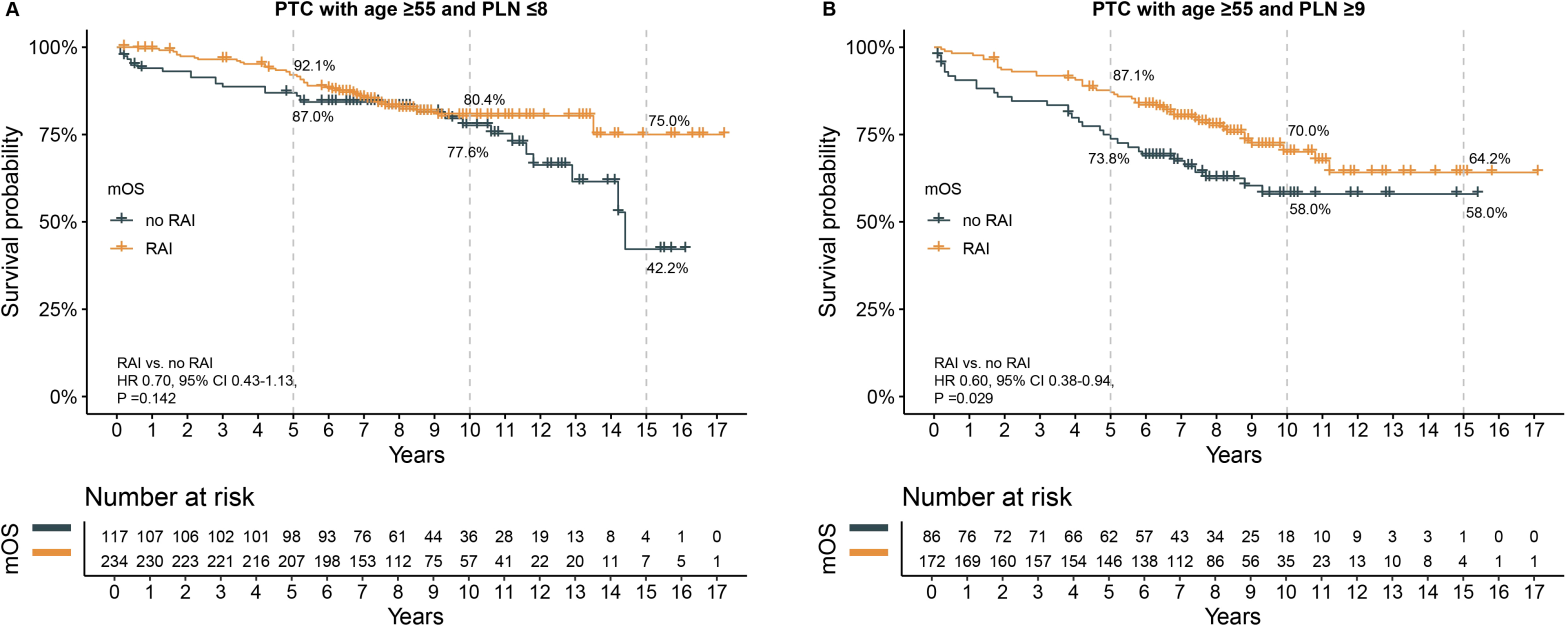
The effect of RAI on survival after propensity score matching in patients with age ≥55 years. **(A)** in subgroup of PLN ≤8, **(B)** in subgroup of PLN ≥9. PTC, papillary thyroid carcinoma; PLN, positive lymph node; RAI, radioactive iodine. HR, hazard ratio; CI, confidence interval.

To adjust for baseline clinical characteristic differences between patients who received RAI and those who did not, PSM was performed. The matched characteristics for the subgroups of PLN ≤8 and PLN ≥9 were shown in **Supplementary Tables S1**, **S2**. No significant differences in clinical characteristics were found post-PSM in either subgroup. In the PLN ≤8 subgroup, survival analysis showed a trend toward better survival in patients who received RAI, although this did not reach statistical significance (HR 0.70, 95% CI 0.43–1.13, P =0.142). The 5-year, 10-year, and 15-year OS rates were higher in the RAI group, with the most notable difference in the 15-year OS rate (75.0% vs. 42.2%) (**Figure 3A**). In the PLN ≥9 subgroup, patients who received RAI had significant better OS, with 5-year (87.1% vs. 73.8%), 10-year (70.0% vs. 58.0%), and 15-year (64.2% vs. 58.0%) OS rates being higher in the RAI group (**Figure 3B**). The HR for death was 0.60 (95% CI 0.38-0.94, P =0.0295).

### The prognostic role of PLN in patients aged <55 years

In patients aged <55 years, no statistically significant cutoff value of PLN was found to stratify the cohort into high-risk and low-risk groups. Therefore, we used the cutoff of PLN ≥9, which had significance in patients aged ≥55 years, to divide the patients into low-risk (PLN ≤8, N =1447) and high-risk (PLN ≥9, N =2012) groups. Similar to the findings in the ≥55-year cohort, patients with PLN ≥9 had a higher proportion of larger tumor sizes (**Table 3**). Additionally, the PLN ≥9 group had a higher proportion of male patients (35.0% vs. 27.5%, P <0.001), while the proportion of female patients was lower (65.0% vs. 72.5%, P <0.001). No differences were observed in the distribution of race or history of RAI therapy between the two groups.

**Table 3:** Clinical characteristics of papillary thyroid carcinoma patients aged <55 years and comparison stratified by positive lymph nodes.

Variable	All, N =3459 (%)	PLN ≤8, N =1447 (%)	PLN ≥9, N =2012 (%)	P value
Sex				<0.001
Male	1102 (31.9)	398 (27.5)	704 (35.0)	
Female	2357 (68.1)	1049 (72.5)	1308 (65.0)	
Race				0.669
White	2856 (82.6)	1202 (83.1)	1654 (82.2)	
Black	80 (2.3)	35 (2.4)	45 (2.2)	
Others	523 (15.1)	210 (14.5)	313 (15.6)	
Tumor size, mm				<0.001
0-10	676 (19.5)	425 (29.4)	251 (12.5)	
11-20	1089 (31.5)	485 (33.5)	604 (30.0)	
21-40	1090 (31.5)	380 (26.3)	710 (35.3)	
>40	503 (14.5)	120 (8.3)	383 (19.0)	
Unknown	101 (2.9)	37 (2.6)	64 (3.2)	
RAI				0.145
No	725 (21.0)	321 (22.2)	404 (20.1)	
Yes	2734 (79.0)	1126 (77.8)	1608 (79.9)	

PLN, positive lymph node; RAI, radioactive iodine.

**Figure 4:**
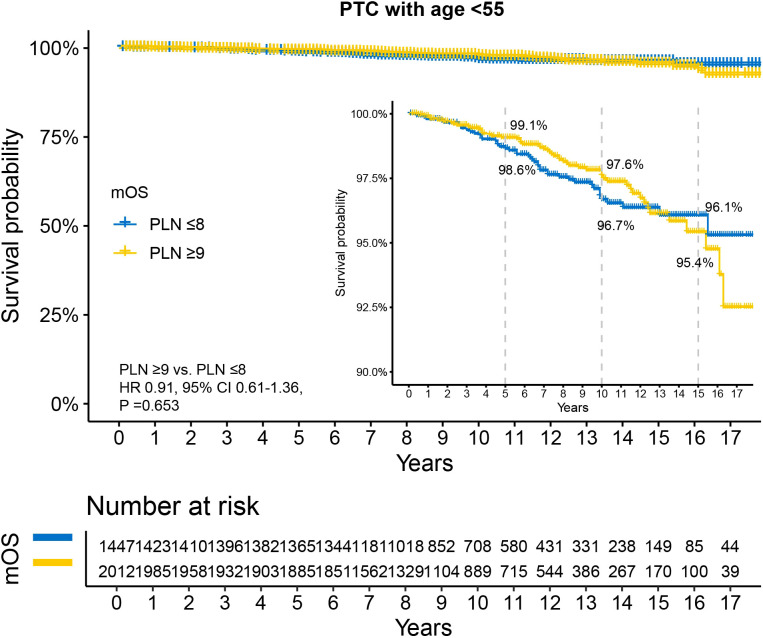
Disparity in survival between PLN ≤8 and PLN ≥9 in N1b PTC patients with age <55 years. All patients underwent total thyroidectomy and at least 10 examined lymph nodes. PTC, papillary thyroid carcinoma; PLN, positive lymph node; HR, hazard ratio; CI, confidence interval.

Kaplan-Meier survival analysis revealed no statistically significant difference in OS between the two groups (HR 0.91, 95% CI 0.61–1.36, P =0.653). The 5-year, 10-year, and 15-year OS rates for patients with PLN ≤8 were 98.6%, 96.7%, and 96.1%, respectively, while those for patients with PLN ≥9 were 99.1%, 97.6%, and 95.4%, respectively (**Figure 4**). Univariable Cox analysis showed that only sex was a significant prognostic factor (**Supplementary Table S3**), with female patients demonstrating better survival (HR 0.24, 95% CI 0.16–0.36, P <0.001).

### The effect of RAI on survival

**Figure 5:**
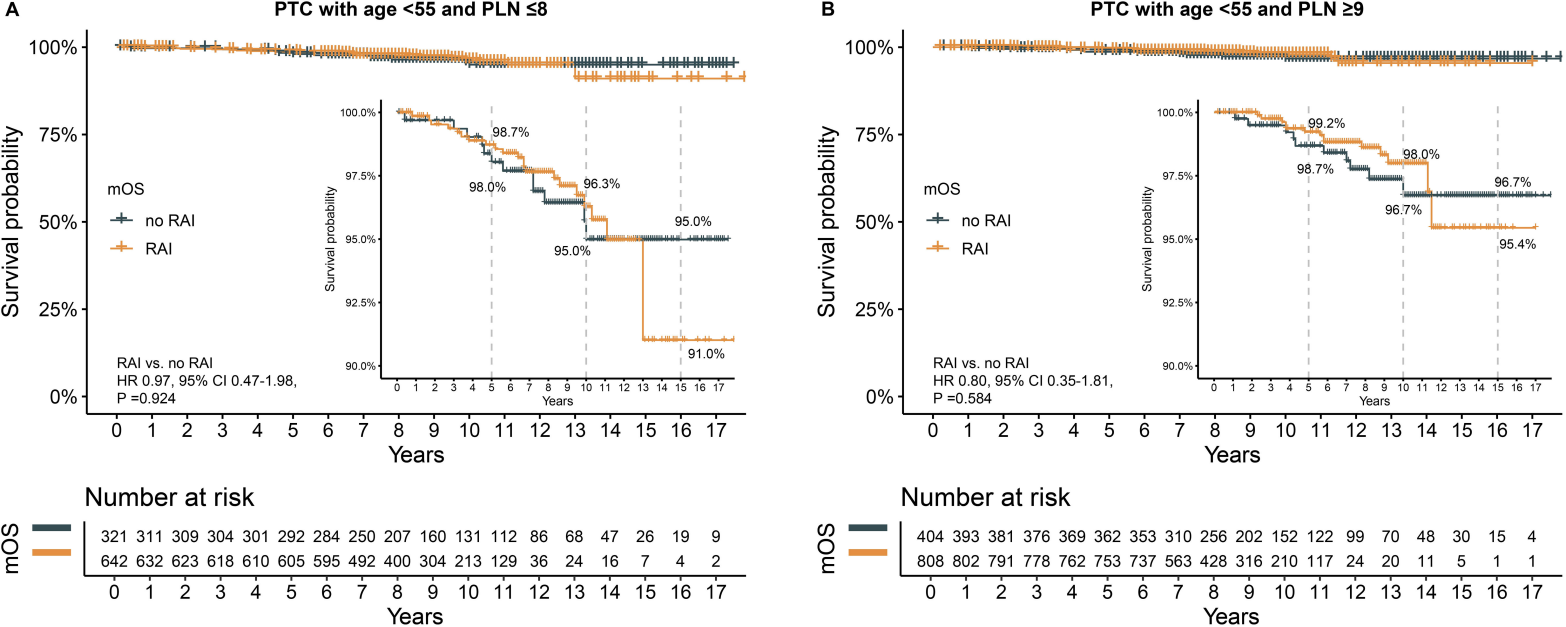
The effect of RAI on survival after propensity score matching in patients with age <55 years. **(A)** in subgroup of PLN ≤8, **(B)** in subgroup of PLN ≥9. PTC, papillary thyroid carcinoma; PLN, positive lymph node; RAI, radioactive iodine. HR, hazard ratio; CI, confidence interval.

After PSM, all clinical characteristics were balanced between the groups, with no significant differences observed (**Supplementary Table S4**, **Supplementary Table S5**). In the subgroup with PLN ≤8, the 5-year (98.7% vs. 98.0%), 10-year (96.3% vs. 95.0%), and 15-year (91.0% vs. 95.0%) OS rates were similar for patients who received RAI and those who did not (**Figure 5A**). The HR for death was not significant (HR 0.97, 95% CI 0.47–1.98, P =0.924).

Similarly, in the subgroup with PLN ≥9, no significant differences were found in 5-year (99.2% vs. 98.7%), 10-year (98.0% vs. 96.7%), and 15-year (95.4% vs. 96.7%) OS rates between the RAI and no RAI groups (**Figure 5B**). The HR for death was also not significant (HR 0.80, 95% CI 0.35–1.81, P =0.584).

## Discussion

This study provided significant insights into the prognostic value of the number of PLN and the role of postoperative RAI therapy in patients with clinical N1b PTC. Our findings revealed that the number of PLN was a critical determinant of long-term survival outcomes. Specifically, patients with ≥9 PLN exhibited significantly poorer survival rates compared to those with fewer PLN. Additionally, postoperative RAI therapy conferred a survival benefit exclusively in patients aged ≥55 years with ≥9 PLN. In contrast, for patients aged ≥55 years with ≤8 PLN, the benefit of RAI therapy remained uncertain. For patients younger than 55 years, no clinical benefit of postoperative RAI therapy was observed.

Several studies had investigated the role of PLN in N1b PTC. For instance, one study found that a higher number of PLN in both the entire neck (HR 1.048, P =0.017) and the lateral compartment (HR 1.095, P =0.037) was associated with recurrence in cN1b PTC patients (12). However, no optimal PLN cutoff had been identified. Another study used a cutoff of PLN =3 to stratify patients with cN1b PTC into two groups, showing an increase in lymph node recurrence but not in residual thyroid recurrence among those with higher PLN (11). While the role of PLN in PTC had been well-established, no prior study had directly examined its impact on long-term survival outcomes in patients with cN1b disease. Our study addressed this gap by directly stratifying N1b PTC patients based on the number of PLN. We demonstrated that the prognostic significance of PLN was particularly evident in patients aged ≥55 years, where ≥9 PLN reliably identified high-risk individuals. The 5-year, 10-year, and 15-year OS rates increased by 6.4%, 11.4%, and 9.2%, respectively, in patients aged ≥55 years with ≤8 PLN compared to those with ≥9 PLN. This underscored the critical impact of LNM on long-term survival in N1b patients aged ≥55 years.

The role of RAI therapy in PTC had remained contentious. Recent studies had suggested that, in low-risk PTC patients, lobectomy might have provided comparable efficacy and long-term survival outcomes to total thyroidectomy combined with RAI, while also reducing postoperative complications (13, 14). Consequently, the 2015 ATA guidelines had recommended selective use of RAI, even in patients with confirmed LNM. However, high-quality evidence to definitively establish when adjuvant RAI therapy was truly necessary remained lacking. In our study, we found that for patients aged ≥55 years with ≥9 PLN, postoperative RAI therapy significantly improved OS at 5 years, 10 years, and 15 years, with increases of 13.3%, 12.0%, and 6.2%, respectively. In contrast, no clinical benefit from postoperative RAI was observed in other subgroups. These findings differed from those of Zhao et al., who also used the SEER database and reported that RAI therapy was associated with improved OS in the entire N1b PTC cohort (8). The discrepancies between the two studies may have been due to the more stringent inclusion criteria applied in our study. For example, we only included patients with at least 10 ELNs, which allowed for a more accurate assessment of LNM status and ensured that decisions regarding RAI therapy were better aligned with the patient’s clinical profile. In Zhao et al.'s study, despite statistical significance for improved OS in the overall N1b cohort, the survival benefit was minimal, particularly for patients with ≥2 metastatic lymph nodes, where the survival benefit for 5-year, 10-year, and 15-year OS rates was less than 5%. Our study, however, further refined the identification of subgroups that truly benefited from postoperative RAI therapy, providing a more precise and scientifically grounded reference for individualized treatment strategies.

Supporting our findings, other studies highlighted age ≥55 years and a lymph node ratio, which was defined as the ratio of positive to examined lymph nodes (PLN/ELN), >0.17 as key factors for selecting patients who would have benefited from post-RAI treatment (15). Wang et al. also observed a survival benefit with RAI in the entire intermediate-risk PTC cohort from the SEER dataset, particularly in older patients using a 45-year age threshold. However, no significant benefit was observed in patients with any N1 disease (16). In contrast, a large institutional study from Korea found no advantage of RAI in preventing locoregional recurrence in intermediate-risk PTC, even in patients with lymph node metastasis (17).

In addition to LNM and age, large tumor size and advanced preoperative tumor staging were identified as high-risk factors influencing the efficacy of postoperative RAI therapy in patients with PTC. Patients with these risk factors were found to benefit more from postoperative RAI, as it reduced the risk of recurrence (18). Moreover, although PTC was typically a well-differentiated tumor, lower tumor differentiation was associated with a compromised response to RAI treatment (18). The effectiveness of postoperative RAI therapy was also closely related to thyroid hormone levels. A study found that changes in thyroglobulin levels after two courses of RAI played a crucial role, with decreases below certain thresholds being associated with worse outcomes (19). Additionally, although the BRAF V600E mutation had been associated with clinical prognosis in PTC, patients with this mutation showed no inferior clinical response to timely postoperative RAI therapy (20, 21). However, the presence of BRAF V600E coupled with TERT promoter mutations, was strongly linked to the loss of RAI avidity and the impairment of iodide-metabolizing machinery in recurrent PTC, demonstrating a robust predictive value for RAI treatment failure in these cases (22). These studies, including our own, emphasized the complexity and variability in the effectiveness of RAI therapy in N1b PTC, underscoring the need to consider patient-specific factors such as age, lymph node involvement, tumor size, and gene mutation, when determining treatment protocols. As more evidence accumulated, an individualized approach to RAI therapy became more refined, offering more targeted and effective management of PTC patients.

### Strength and limitation

This study provided important insights into the prognostic value of the number of PLN in patients with N1b PTC, highlighting it as a significant factor influencing survival outcomes. In addition, we identified a subgroup who might have benefited the most from postoperative RAI therapy, suggesting a more personalized approach to comprehensive treatment, and indicating that not all patients with N1b PTC required RAI therapy.

There were several limitations to be acknowledged. First, the retrospective design introduced potential biases. We minimized the impact of unknown biases on the results using multivariate Cox and PSM analyses. Second, patients with fewer than 10 ELNs were excluded from this study, which may limit the applicability of our findings to clinical practice where limited lymph node dissection is performed. Third, the treatment protocols, including the dosage and time of RAI, were heterogeneous across institutions and clinicians from 17 registries, which limited the ability to make a clearer recommendation of postoperative RAI protocol for selected subgroup. Fourth, important pathological features—such as the size of lymph node metastases and the presence of extranodal extension—were not available in the SEER database. These variables may influence prognosis and treatment decisions, but could not be assessed in our analysis. Finally, the study's focus on a single database of N1b PTC patients may have limited the generalizability of the findings to broader populations. Additional research was needed to validate these results in more diverse and larger patient cohorts.

## Conclusion

The number of PLN as a key prognostic factor for survival in resected N1b PTC patients. More importantly, our findings suggested that not all N1b PTC patients required postoperative RAI therapy, and we identified a subgroup of patients (age ≥55 years and PLN ≥9) who were more likely to benefit from it.

## Data Availability

The raw data supporting the conclusions of this article will be made available by the authors, without undue reservation.
