# Supplementary material for: Impact of positive lymph nodes and RAI therapy on survival in N1b papillary thyroid carcinoma
Source: Front Endocrinol (Lausanne). 2025 May 22;16:1551075. doi: 10.3389/fendo.2025.1551075 (PMC12137069; doi:10.3389/fendo.2025.1551075)
Supplement: Supplementary file 1 [file Table1.docx]

Table S1: Clinical characteristics comparison between RAI group and no RAI group in PTC patients aged ≥55 years and PLN ≤8.

| **Variable** | **All,**  **N =351 (%)** | **RAI,**  **N =234 (%)** | **no RAI,**  **N =117 (%)** | **P value** |
| --- | --- | --- | --- | --- |
| **Sex** |  |  |  | 0.676 |
| Male | 196 (55.8) | 133 (56.8) | 63 (53.8) |  |
| Female | 155 (44.2) | 101 (43.2) | 54 (46.2) |  |
| **Race** |  |  |  | 0.191 |
| White | 297 (84.6) | 200 (85.5) | 97 (82.9) |  |
| Black | 10 (2.8) | 4 (1.7) | 6 (5.1) |  |
| Others | 44 (12.5) | 30 (12.8) | 14 (12.0) |  |
| **Tumor size, mm** |  |  |  | 1 |
| 0-10 | 140 (39.9) | 94 (40.2) | 46 (39.3) |  |
| 11-20 | 78 (22.2) | 52 (22.2) | 26 (22.2) |  |
| 21-40 | 69 (19.7) | 46 (19.7) | 23 (19.7) |  |
| >40 | 52 (14.8) | 34 (14.5) | 18 (15.4) |  |
| Unknown | 12 (3.4) | 8 (3.4) | 4 (3.4) |  |

RAI, radioactive iodine; PTC, papillary thyroid carcinoma; PLN, positive lymph node.

Table S2: Clinical characteristics comparison between RAI group and no RAI group in PTC patients aged ≥55 years and PLN ≥9.

| **Variable** | **All,**  **N =258 (%)** | **RAI,**  **N =172 (%)** | **no RAI,**  **N =86 (%)** | **P value** |
| --- | --- | --- | --- | --- |
| **Sex** |  |  |  | 0.825 |
| Male | 139 (53.9) | 94 (54.7) | 45 (52.3) |  |
| Female | 119 (46.1) | 78 (45.3) | 41 (47.7) |  |
| **Race** |  |  |  | 0.561 |
| White | 213 (82.6) | 145 (84.3) | 68 (79.1) |  |
| Black | 3 (1.2) | 2 (1.2) | 1 (1.2) |  |
| Others | 153 (17.3) | 81 (15.5) | 72 (19.9) |  |
| **Tumor size, mm** |  |  |  | 0.998 |
| 0-10 | 34 (13.2) | 22 (12.8) | 12 (14.0) |  |
| 11-20 | 64 (24.8) | 43 (25.0) | 21 (24.4) |  |
| 21-40 | 104 (40.3) | 70 (40.7) | 34 (39.5) |  |
| >40 | 42 (16.3) | 28 (16.3) | 14 (16.3) |  |
| Unknown | 14 (5.4) | 9 (5.2) | 5 (5.8) |  |

RAI, radioactive iodine; PTC, papillary thyroid carcinoma; PLN, positive lymph node.

Table S3: Univariable and multivariable Cox analyses for risk factors associated with overall survival in patients aged <55 years.

| **Variable** | **Univariable** | | **Multivariable** | |
| --- | --- | --- | --- | --- |
|  | **HR (95% CI)** | **P value** | **HR (95% CI)** | **p value** |
| **Sex** |  |  |  |  |
| Male | Reference | - |  |  |
| Female | 0.24 (0.16-0.36) | <0.001 |  |  |
| **Race** |  |  |  |  |
| White | Reference | - |  |  |
| Black | 0.88 (0.22-3.57) | 0.854 |  |  |
| Others | 0.80 (0.44-1.47) | 0.473 |  |  |
| **Tumor size, mm** |  |  |  |  |
| 0-10 | Reference | - |  |  |
| 11-20 | 0.57 (0.32-1.03) | 0.062 |  |  |
| 21-40 | 0.75 (0.43-1.30) | 0.310 |  |  |
| >40 | 1.18 (0.65-2.16) | 0.579 |  |  |
| Unknown | 1.06 (0.36-3.05) | 0.920 |  |  |
| **RAI** | |  |  |  |
| No | Reference | - |  |  |
| Yes | 0.84 (0.52-1.34) | 0.458 |  |  |
| **PLN** |  |  |  |  |
| ≤8 | Reference | - |  |  |
| ≥9 | 0.91 (0.61-1.36) | 0.653 |  |  |

HR, hazard ratio; CI, confidence interval; RAI, radioactive iodine; PLN, positive lymph node.

Table S4: Clinical characteristics comparison between RAI group and no RAI group in PTC patients aged <55 years and PLN ≤8.

| **Variable** | **All,**  **N =963 (%)** | **RAI,**  **N =642 (%)** | **No RAI,**  **N =321 (%)** | **P value** |
| --- | --- | --- | --- | --- |
| **Sex** |  |  |  | 1 |
| Male | 220 (22.8) | 147 (22.9) | 73 (22.7) |  |
| Female | 743 (77.2) | 495 (77.1) | 248 (77.3) |  |
| **Race** |  |  |  | 0.784 |
| White | 806 (83.7) | 538 (83.8) | 268 (83.5) |  |
| Black | 17 (1.8) | 10 (1.6) | 7 (2.2) |  |
| Others | 140 (14.5) | 94 (14.6) | 46 (14.3) |  |
| **Tumor size, mm** |  |  |  | 1 |
| 0-10 | 291 (30.2) | 193 (30.1) | 98 (30.5) |  |
| 11-20 | 308 (32.0) | 206 (32.1) | 102 (31.8) |  |
| 21-40 | 274 (28.5) | 183 (28.5) | 91 (28.3) |  |
| >40 | 63 (6.5) | 42 (6.5) | 21 (6.5) |  |
| Unknown | 27 (2.8) | 18 (2.8) | 9 (2.8) |  |

RAI, radioactive iodine; PTC, papillary thyroid carcinoma; PLN, positive lymph node.

Table S5: Clinical characteristics comparison between RAI group and no RAI group in PTC patients aged <55 years and PLN ≥9.

| **Variable** | **All,**  **N =1212 (%)** | **RAI,**  **N =808 (%)** | **No RAI,**  **N =404 (%)** | **P value** |
| --- | --- | --- | --- | --- |
| **Sex** |  |  |  | 0.916 |
| Male | 436 (36.0) | 292 (36.1) | 144 (35.6) |  |
| Female | 776 (64.0) | 516 (63.9) | 260 (64.4) |  |
| **Race** |  |  |  | 0.617 |
| White | 1015 (83.7) | 679 (84.0) | 336 (83.2) |  |
| Black | 26 (2.1) | 15 (1.9) | 11 (2.7) |  |
| Others | 171 (14.1) | 114 (14.1) | 57 (14.1) |  |
| **Tumor size, mm** |  |  |  | 0.983 |
| 0-10 | 150 (12.4) | 99 (12.3) | 51 (12.6) |  |
| 11-20 | 361 (29.8) | 241 (29.8) | 120 (29.7) |  |
| 21-40 | 372 (30.7) | 248 (30.7) | 124 (30.7) |  |
| >40 | 275 (22.7) | 182 (22.5) | 93 (23.0) |  |
| Unknown | 54 (4.5) | 38 (4.7) | 16 (4.0) |  |

RAI, radioactive iodine; PTC, papillary thyroid carcinoma; PLN, positive lymph node.
